# Clinical application of cervical shear wave elastography in predicting the risk of preterm delivery in DCDA twin pregnancy

**DOI:** 10.1186/s12884-022-04526-0

**Published:** 2022-03-14

**Authors:** Jimei Sun, Nan Li, Wei Jian, Dingya Cao, Junying Yang, Min Chen

**Affiliations:** 1 Department of Obstetrics and Gynecology, Department of Fetal Medicine and Prenatal Diagnosis, Key Laboratory for Major Obstetric Diseases of Guangdong Province, The Third Affiliated Hospital of Guangzhou Medical University, 63 Duobao Road, Liwan District, Guangzhou, China; 2 Global UIS Academic Department, Shenzhen Mindray Bio-Medical Electronics Co., Ltd, Shenzhen, China

**Keywords:** Shear wave elastography, Preterm delivery, Twin pregnancy, Dichorionic Diamniotic

## Abstract

**Background:**

Limited studies have used cervical shear wave elastography (SWE) as a tool to investigate the predictive effect of cervical changes on preterm delivery (PTD) in twin pregnancy. This study is aimed to predict the risk of PTD by cervical SWE in dichorionic diamniotic (DCDA) twin pregnancy.

**Methods:**

A total of 138 women with dichorionic diamniotic (DCDA) twins were included in this prospective study. The mean SWE value of the cervix was obtained from the inner, middle and outer regions of the anterior and posterior cervical lips using a transvaginal ultrasound transducer and measured consecutively across three different gestations (20–23^+ 6^ weeks, 24–27^+ 6^ weeks, and 28–32 weeks). Follow-up was performed on all subjects, and we compared the mean SWE value between the PTD and term delivery (TD) groups.

**Results:**

A total of 1656 cervical mean SWE data were collected for analysis. Among the 138 twin pregnant women, only 92 women completed the three elastography examinations; PTD occurred in 58.7% (54/92), and TD in 41.3% (38/92). The mean (SD) maternal age was 33.1 ± 4.1 years, and the mean (SD) body mass index was 21.1 ± 2.6 kg/m^2^. As gestational age increased, the mean SWE value of each part of the cervix decreased. The cervical mean SWE value was lower in the preterm group than in the term group in all three gestations, except for the anterior cervical lip at 28–32 weeks. Receiver operating characteristics (ROC) curves showed the sensitivity of mean SWE value of the anterior cervical lip was 83.3% (95% CI, 70.7–92.1) with a specificity of 57.9% (95% CI, 40.8–73.7) for predicting PTD at a cutoff value of 7.94 kPa. The positive likelihood ratio (LR+) was 1.67 (95% CI, 1.19–2.34), and the negative likelihood ratio (LR–) was 0.33 (95% CI, 0.17–0.64).

**Conclusions:**

There is a significant negative correlation between cervical stiffness and gestational age in DCDA twin pregnancy. SWE is a potential tool for assessing cervical stiffness and predicting PTD in DCDA twin pregnancy.

**Supplementary Information:**

The online version contains supplementary material available at 10.1186/s12884-022-04526-0.

## Introduction

The development and broad application of assisted reproductive technology in recent years and the increased prevalence of advanced maternal age have led to a rising incidence of twin pregnancies worldwide. Preterm delivery (PTD), defined as birth before 37 weeks of pregnancy, is a significant public health problem. The risk of PTD between 24 and 34 weeks of gestation is 59% in monochorionic twins and 54% in dichorionic twins [1]. PTD is the major perinatal complication accounting for about 50% of the complications associated with twin pregnancies [2, 3]. It is associated with short-term and long-term consequences, such as a higher risk of neurodevelopmental impairment, respiratory and gastrointestinal complications, blindness, deafness, mental and behavioral deficits, and chronic disorders in adulthood [4–8]. The neonatal morbidity and mortality resulting from PTD impose a substantial economic burden on health services worldwide. Several methods are currently being used to predict preterm labor in singleton pregnancies, including taking the history of previous preterm birth or late miscarriage, measurement of cervical length, fetal fibronectin, inflammatory markers, and the vaginal microbiome [9, 10]. However, the prediction of PTD in twin pregnancy has been rarely investigated because multiple pregnancies encompass various underlying pathological mechanisms [11]. Identifying patients at higher risk of PTD in twin pregnancies would help develop effective interventions to prevent adverse perinatal outcomes associated with PTD. In addition, risk assessment of PTD can avoid unnecessary interventions for lower-risk women and save medical service resources.

Although there are multiple causes of spontaneous PTD, three main factors that lead to spontaneous PTD include cervical remodeling, premature myometrial contractions, and premature rupture of membranes [12]. These events can occur in no particular sequence but may interactively affect each other [13]. Cervical remodeling is a process of ripening characterized by softening, disappearance, and finally early dilation of the cervix. The function of the cervix is to keep the fetus in the mother’s uterus during the softening process and facilitate birth after it ripens and dilates [14]. Premature softening and ripening of the cervix are related to shortening and PTD. Currently, various methods have been suggested for predicting PTD in twin pregnancies, and transvaginal cervical length (TVCL) measurement in the second trimester has been shown to help predict spontaneous PTD [15–17]. However, the major limitation of using cervical length alone in the second trimester is its low sensitivity and positive predictive value for PTD [16, 18].

It would be beneficial to consider other earlier complementary PTD prediction methods to provide prevention promptly. Transvaginal cervical elastography is a non-invasive and repeatable screening technique for measuring cervical softness in pregnant women. Elastography methods can be classified into strain elastography (SE) and shear wave elastography (SWE) based on the physical parameters being measured [19]. However, SE requires the application of pressure on the ultrasound probe to generate a ‘tissue deformation’ on the region of interest (ROI) [20]. In addition, the manual force applied to the probe depends on the hardness of the cervical tissue. The harder the cervix is, the more pressure is required to compress it and vice versa [19, 20]. Since the magnitude of pressure is highly influenced by the operator, SE is not an objective nor a standardized method. In contrast, SWE relies on acoustic radiation force impulse for imaging and measurements and is operator-independent [21]. SWE is a non-invasive approach that quantifies tissue mechanical properties, such as stiffness, using shear wave speed in the target tissue [22, 23]. Recently, it has been widely performed on patients with varying degrees of liver fibrosis [24], benign and malignant nodules in the breast and thyroid [25, 26] and the pathological staging of prostate cancer [27].

Several studies have investigated the role of SWE in predicting PTD or labor induction of singleton pregnancy, but very few have focused on twin pregnancy [28–31]. Since DCDA pregnancy is associated with lower rates of miscarriage and preterm birth than monochorionic diamniotic (MCDA) pregnancy, we performed cervical SWE on women with DCDA twins to investigate the changes in cervical tissue stiffness during three gestational periods and to assess the predictive value of SWE for PTD before 37 weeks in DCDA twin pregnancy.

## Material and methods

### Subjects

This study was conducted between August 2020 and June 2021. Eligible Chinese pregnant women carrying DCDA twins were invited to participate in this study and were enrolled at 20–23^+ 6^ weeks of gestation from The Third Affiliated Hospital of Guangzhou Medical University. All cervical SWE measurements on each woman were performed by one operator (JM Sun). Cervical length and stiffness (by SWE) were measured across three consecutive gestations (20–23^+ 6^ weeks, 24–27^+ 6^ weeks and 28–32 weeks) until spontaneous labor or admission for induction of labor. Women aged less than 18 years and women who had cervical lesions, uterine malformations or cervical surgery history were excluded. Demographic data, obstetric data and pregnancy outcomes were collected. The study protocol was reviewed and approved by the Institution Ethics Committee of the Third Affiliated Hospital of Guangzhou Medical University (approval number: 2020172). Written informed consent was obtained from all subjects.

### Measurement of cervical elasticity by SWE

Before the examination, all pregnant women were put in a lithotomy position with an empty bladder. A transvaginal scan of the cervix was performed using a Resona R9 system (Mindray Medical International, Shenzhen, China) equipped with a DE10-3WU endovaginal probe. Measurement of cervical length and cervical stiffness by SWE were performed by one operator who has been accredited for the measurement of cervical length by the Fetal Medicine Foundation (FMF) (www.fetalmedicine.org) and underwent extensive cervical elastography training. Two operators assessed the measurements of the first 30 women to evaluate the intra- and inter-observer reproducibility. Both operators were blinded to the images assessed by each other.

The Resona R9 system provides the motion stability index (M-STB Index), ranging from one to five green stars, to help physicians monitor the stability of the shear wave on cervix tissue in real-time. The lower the motion interference, the more the green stars. When the M-STB index is four or five stars, it indicates that limited tissue motion has little influence on the shear wave stability in the ROI. The system also uses the reliability index (RLB-index) for additional quality control. RLB Map reflects the credible and non-credible regions of the data results on a shear wave image in real-time. The green region is highly credible, whereas the purple region is of low credibility. When the RLB map is uniformly distributed green, and the RLB-index is over 95%, it shows high credibility.

**Fig. 1:**
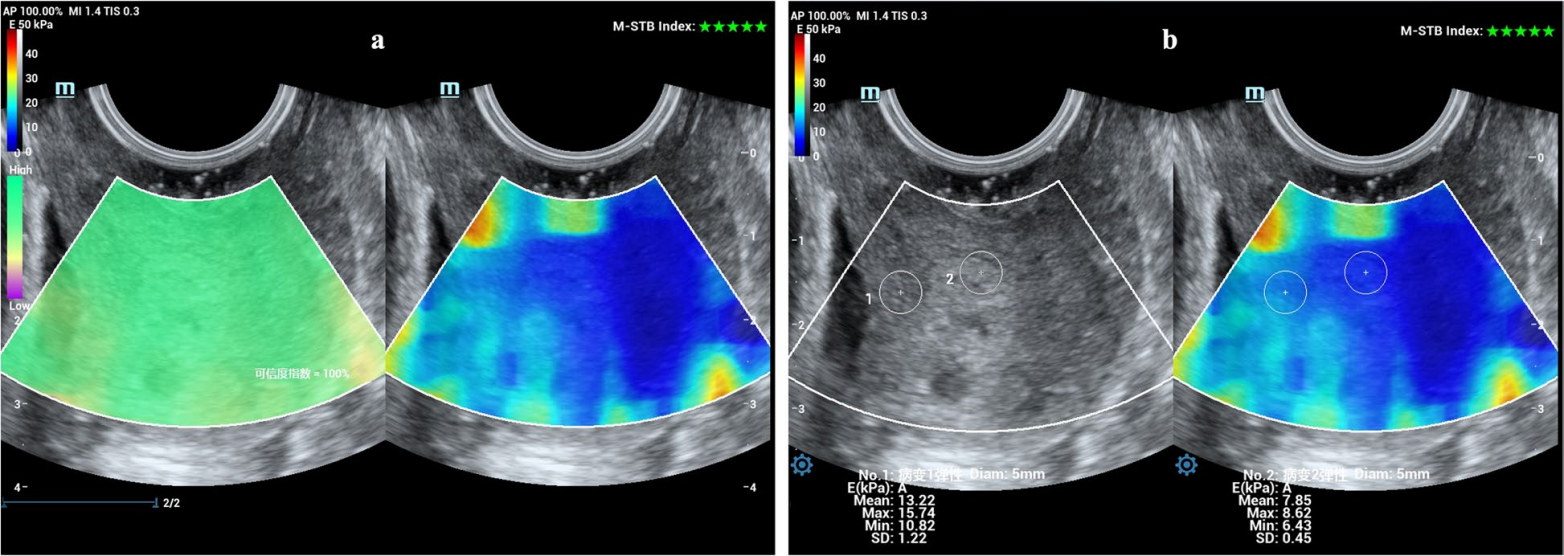
Measurement of cervical mean SWE value. Cervical stiffness of the inner, middle and outer regions of the anterior and posterior cervical lip was measured using SWE. Inner anterior cervical lip (ACL), inner posterior cervical lip (PCL); middle ACL, middle PCL; outer ACL, outer PCL. The diameter of the target regions of interest was set to 5 mm

Cervical length was measured in strict compliance with the FMF protocol. Once cervical length measurement was completed, the operator turned on the cervical shear wave software and generated an elastography map of the entire cervix. Three cervical elastography images were acquired when the shear wave images became stable, which RLB-index was>95%, and the M-STB index had four stars or above (Fig. 1a). The mean SWE value of the cervix was obtained from six regions, namely the inner, middle and outer regions of the anterior and posterior cervical lips with a 5-mm-diameter ROI (Fig. 1b). For subjects in whom the entire elastography cervix map was difficult to obtain, the cervical elastography images of the ROIs were acquired separately. The Young’s modulus value (E) is related to the shear wave speed (Vs): E = 3ρVs^2^, where ρ represents a density of the tissue (1000 kg/m3). The tissue stiffness measured by SWE can be expressed as either shear wave speed in m/s or Young’s modulus in kPa [32]. The machine automatically calculates the mean SWE value into Young’s modulus and shows it on the lower screen in “kPa” (Fig. 1b). The cervical stiffness of the participants was measured using the same method across three consecutive gestations (20–23^+ 6^ weeks, 24–27^+ 6^ weeks and 28–32 weeks).

#### Statistical analysis

The cervical stiffness and baseline characteristics were compared between the preterm and the term pregnancy groups. The normality of variables was assessed using the Kolmogorov–Smirnov test. Normally distributed continuous variables and non-normally distributed variables were analyzed using the Student’s t-test and Mann-Whitney U test. Categorical variables were compared using the chi-square test or Fisher’s exact test. The intra- and inter-observer agreements were assessed by the intraclass correlation coefficient (ICC). A binary logistic regression analysis was used to calculate the significant covariates for predicting PTD. ROC curves were then plotted for the regression models to evaluate their discriminative ability. Two-way repeated-measures ANOVA was used to compare the stiffness of different regions of the cervical lip in different gestational ages. A two-tailed *P* value of < 0.05 was considered statistically significant. Statistical analysis was performed using IBM SPSS version 23 (SPSS, Inc., Chicago, IL, USA) and MedCalc Statistical Software version 20.0.1 (MedCalc Software Ltd., Ostend, Belgium).

#### Ethics

The study protocol was reviewed and approved by the Institution Ethics Committee of the Third Affiliated Hospital of Guangzhou Medical University (approval number: 2020172). Written informed consent was obtained from all participants.

## Results

**Fig. 2:**
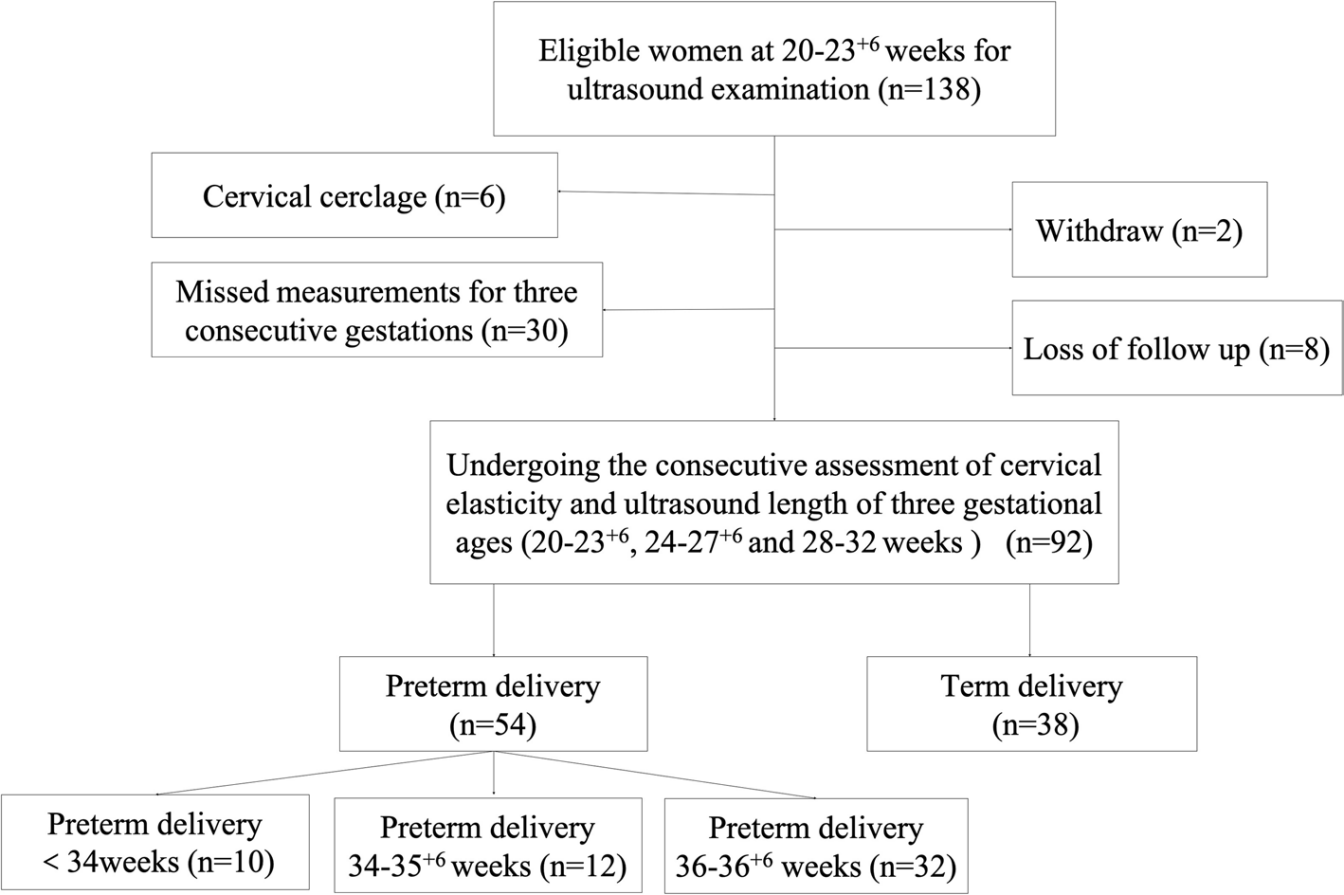
Study workflow

Among 138 women, six were excluded due to cervical cerclage, two withdrew, eight were excluded due to loss of follow-up, and 30 had incomplete serial measurements. The remaining 92 women completed the three elastography examinations, and 1656 cervical elasticity data were collected for subsequent analysis. There were 54 cases of PTD, including 10 cases before 34 weeks, 12 cases between 34 and 35^+ 6^ weeks, and 32 cases between 36 and 36^+ 6^ weeks. The prevalence of PTD and TD was 58.7% (54/92) and 41.3% (38/92), respectively (Fig. 2).

Baseline characteristics of pregnant women and the outcome of neonates are summarized in Table 1. There were significant differences in gestational age at birth, birth weight and neonatal intensive care unit (NICU) admission. However, no differences in maternal age, BMI, obstetric history, prior history of PTD, the way of conception and Apgar score between the two groups. The median gestation at birth was significantly lower in the PTD group [36.14 (34.25, 36.71) weeks] than in the TD group [37.29 (37.11, 37.61) weeks] (*p* < 0.001). The median birth weight was higher in the TD group than in the PTD group [twin 1: 2632.5 (2437.5, 2632.5)g vs. 2385 (1972.5, 2550)g, *P*<0.001; twin 2: 2620 (2450, 2802.5)g vs. 2285 (2025.75, 2442.5)g, *P* = 0.001]. The PTD group had more newborns admitted to NICU than TD group [twin 1 NICU admission: 17 (31.5%) vs. 0(0%), *P*<0.001; twin 2 NICU admission: 17 (31.5%) vs. 2 (5.3%), *P* = 0.003].

**Table 1:** Baseline characteristics of pregnant women and the outcome of neonates

Characteristics	Preterm delivery (*n*=54)	Term delivery (*n*=38)	*p*-value
maternal age	32.5 (30–35)	33 (31–35)	0.496
BMI (kg/m^2^)	22.05 (18.95–22.6)	20.4 (19.55–23.8)	0.671
parous women	16 (29.6)	15(39.5)	0.325
prior history of PTD	3 (5.6)	4(10.5)	0.39
The way of conception			0.067
Natural conception	1 (1.9)	6 (15.8)	
IVF	45 (83.3)	26 (68.4)	
IUI	1 (1.9)	0 (0)	
Ovarian stimulation	7 (13)	6 (15.8)	
gestational age at birth	36.14 (34.25–36.71)	37.29 (37.11–37.61)	<0.001
twin 1 weight (g)	2385 (1972.5–2550)	2632.5 (2437.5–2632.5)	<0.001
twin 2 weight (g)	2285 (2025.75–2442.5)	2620 (2450–2802.5)	0.001
twin 1 NICU admission	17(31.5)	0(0)	<0.001
twin 2 NICU admission	17(31.5)	2(5.3)	0.003
twin 1 Apgar score < 7 at 1 min	1(1.9)	0(0)	>0.999
twin 2 Apgar score < 7 at 1 min	1(1.9)	0(0)	>0.999

Data are presented as the median (range) or number (%)

*BMI* Body mass index, *IVF* In vitro fertilization, *IUI* Intrauterine insemination, *NICU* Neonatal intensive care unit

**Fig. 3:**
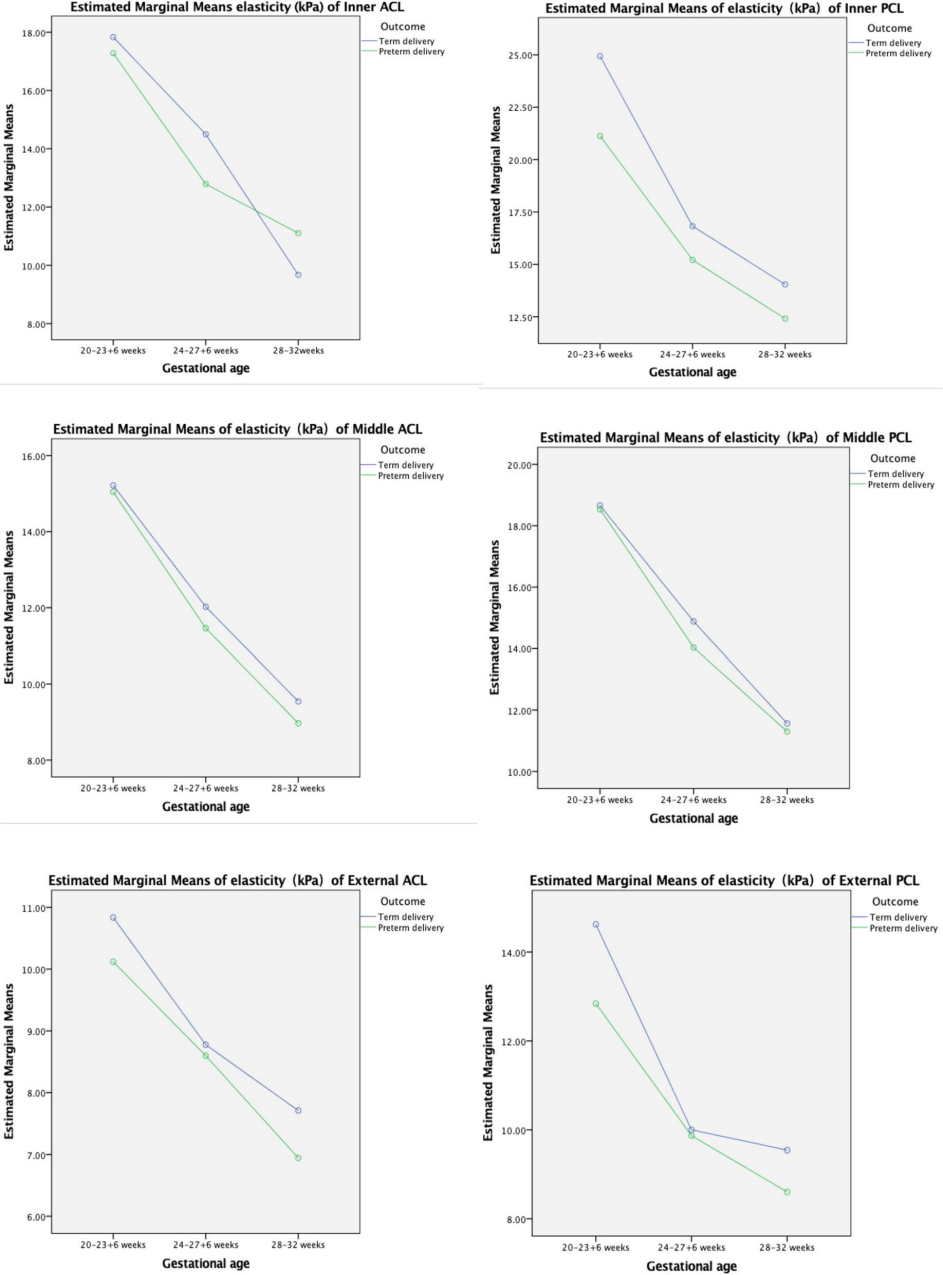
The cervical mean SWE value significantly decreases as gestational age increases

**Fig. 4:**
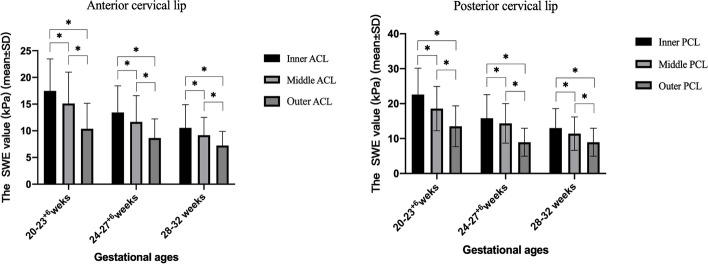
The cervical mean SWE value decreases from the inner region to the outer region of both the anterior and posterior cervical lip during pregnancy

We compared the mean SWE value of the six cervical regions across three gestational ages between the TD and PTD groups (Fig. 3). Cervical softness was significantly highest at 20–23^+ 6^ weeks (*p* < 0.001) (Fig. 3). In addition, the cervical mean SWE value was lower in the PTD group than in the TD group in all three gestational ages, except for the anterior cervical lip at 28–32 weeks. The cervical mean SWE value also decreased from the inner os to the outer os of the anterior and posterior cervical lips during pregnancy (Fig. 4).

**Fig. 5:**
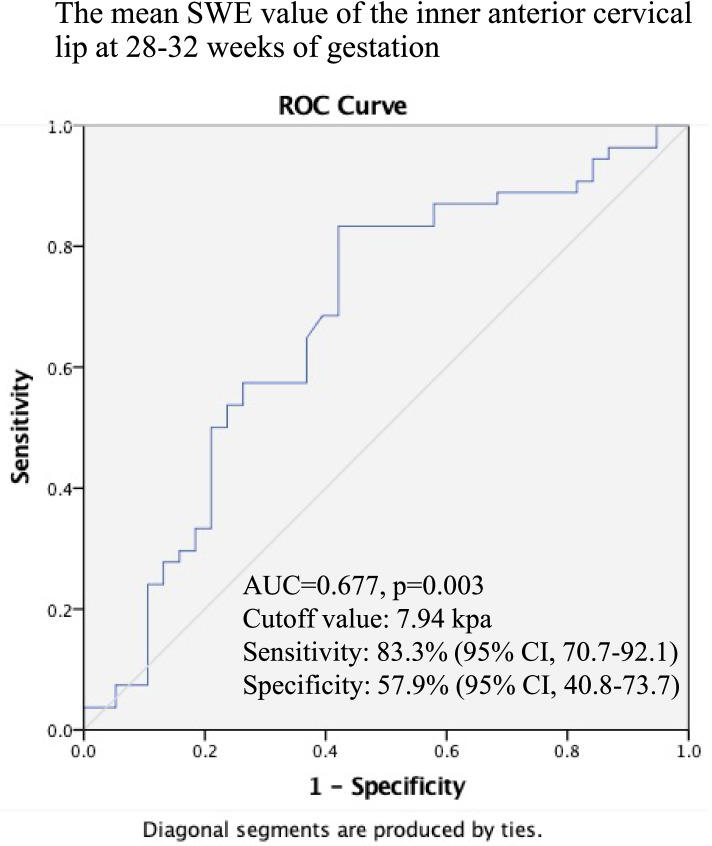
The ROC curve of the mean SWE value of the inner anterior cervical lip at 28–32 weeks of gestation

The receiver operating characteristic (ROC) curve analysis showed that the inner anterior cervical lip (ACL) at 28–32 weeks had the highest AUC (AUC = 0.677; *p* = 0.003; 95% confidence interval [CI], 0.571–0.771) than other parameters (Fig. 5, Supplemental Fig. 1). The sensitivity and specificity of the inner ACL were 83.3% (95% CI, 70.7–92.1) and 57.9% (95% CI, 40.8–73.7) when the cutoff value was 7.94kpa with a positive likelihood ratio (LR+), 1.67 (95% CI, 1.19–2.34) and negative likelihood ratio (LR–), 0.33 (95% CI, 0.17–0.64) (Fig. 5). Cervical length was a poor predictor for twin pregnancy. Its AUC was 0.508 (95% CI, 0.388–0.628) at 20–23^+ 6^ weeks, 0.556 (95% CI, 0.437–0.675) at 24–27^+ 6^ weeks, and 0.540 (95% CI, 0.421–0.659) at 28–32 weeks, respectively (Supplemental Fig. 2).

The intra- and inter-observer agreements were assessed using two sonographers’ intraclass correlation coefficient (ICC). The ICC of each ROI was > 0.88 and > 0.85 for intra- and inter-observer agreement, respectively (Supplemental Table 1).

## Discussion

The findings of this study suggest that the mean SWE value of the cervical lip may improve the prediction of PTD and can be used as a complementary screening method for cervical length in DCDA twin pregnancy. The mean SWE value at 28–32 weeks of the inner ACL is independently associated with PTD of DCDA twins and is superior to the cervical length measurement. There are several possible reasons for the higher predictive value of ACL elasticity than other parameters. Firstly, the internal os of the cervix has a sphincter-like structure in which smooth muscle tissues are concentrated around the cervix opening [33]. The internal os is essential for maintaining the normal shape of the cervical tissue and adjusting its length. Secondly, the SWE value of the ACL is a more confident measurement than that of the posterior cervical lip (PCL) because shear wave propagation is limited at further depth, resulting in higher shear wave loss in the PCL. Finally, the elasticity of the internal os is the highest compared to other regions. When the inner cervix is subjected to increased pressure from the uterine cavity and the pulling force of the cervix itself, the hardening of the internal cervix may serve as a protective mechanism for PTD.

According to the previous studies [31, 34, 35], cervical stiffness is heterogeneous and gradually decreases from the inner region to the outer region of the cervix, which is consistent with our study. In addition, our findings showed that as gestational age increased, the cervix gradually became softer. The human cervix is a dynamic structure composed of smooth muscles, fibroblasts, blood vessels and epithelial tissues, which form a fibrous connective tissue network consisting of 70% type I collagen and 30% type III collagen proteoglycan and elastin [36–38]. Joy et al. reported that the cervical mechanical strength in pregnancy depends on two aspects of cervical tissue architecture, the type of collagen network and degree of tissue hydration [12]. Due to cervical remodeling during pregnancy, the cervix tissue is softened at distinct stages by increasing instability and solubility of the collagen cross-links. In some rodent models, the spacing between the collagen and elastic fiber increases and mass density changes as pregnancy progresses [39].

Compared with the TD group, the PTD group has lower SWE values for all six cervical ROIs in the three gestational ages, except for the inner ACL at 28–32 weeks. Furthermore, cervical SWE value decreases as gestational age increases.

Many studies have used cervical length, cervical elastography or cervical consistency index to identify singleton pregnancy at risk of PTD. In twin pregnancies, most studies have focused on cervical length as a predictor for PTD [18, 40–43], very few have used cervical SWE as a tool to investigate the predictive effect of cervical changes on PTD. Ono et al. reported that SWE is a helpful tool for measuring the changes in cervical stiffness in 254 singleton pregnancies and 26 twin pregnancies [23]. Diawtipsukon et al. proposed that cervical SWE may be an additional option for monitoring the change in cervical softness in twin pregnancies by detecting the difference in cervical softness between singleton and twin pregnancies [44]. However, our study differs from these studies in three issues:

1. These studies had 2 ROIs consisting of the upper and lower regions of the ACL, whereas our study had 6 ROIs encompassing different regions of both the anterior and posterior cervical lip.
2. Previous studies included measurements in the first trimester while we began the cervical measurement at 20 weeks and performed repeated measurements across three consecutive gestational ages.
3. These studies only had 26 or 36 women with twin pregnancies, while our study had a much larger sample size.

The main strength of our study is the prospective design, with three consecutive measurements of cervical elasticity across three consecutive gestations to analyze the trend of change in cervical stiffness and the association between stiffness and PTD in DCDA twin pregnancy. To the best of our knowledge, this is the first study that applied SWE to predict PTD in DCDA twin pregnancy with relatively large sample size. However, there are also several limitations in this study. First, this study was conducted single-center, and there may be bias. Second, the study population consisted of only Chinese women, which may limit generalizability to other populations. Finally, a systematic review involving 29,864 twin pregnancies showed that the perinatal morbidity and mortality of twins differ by chorionicity, and monochorionic twin pregnancies are significantly associated with an increased risk of PTD [45]. However, our analysis only included DCDA twin pregnancy.

In conclusion, our study demonstrated a significant negative correlation between cervical stiffness and gestational age in DCDA twin pregnancy. Cervical SWE value is lower in the PTD group than TD across all three gestational ages, except for the ACL at 28–32 weeks. In addition, SWE value decreases from the inner os to the outer os of the cervix in all three gestational ages. SWE is a potential tool for assessing cervical stiffness and predicting PTD in DCDA twin pregnancy.

## Supplementary Information

**Additional file 1: Supplemental Table 1.** Intra-class correlation coefficients of intra- and inter-observer agreement at six ROIs.

**Additional file 2: Supplemental Figure 1.** The ROC curves of the mean SWE value of the different cervical ROIs.

**Additional file 3: Supplemental Figure 2.** The ROC curves of the cervical length.

## Data Availability

The datasets generated during the current study are available from the corresponding author on reasonable request. The authors may not be able to make their datasets publicly available to protect patients’ privacy.

## Abbreviations

SWE: Shear wave elastography
PTD: Preterm delivery
DCDA: Dichorionic diamniotic
TD: Term delivery
ROC: Receiver operating characteristic
SE: Strain elastography
ROI: Region of interest
MCDA: Monochorionic diamniotic
FMF: Fetal Medicine Foundation
ICC: Intraclass correlation coefficient
ACL: Anterior cervical lip
PCL: Posterior cervical lip
