# Supplementary material for: Clinical application of cervical shear wave elastography in predicting the risk of preterm delivery in DCDA twin pregnancy
Source: BMC Pregnancy Childbirth. 2022 Mar 14;22:202. doi: 10.1186/s12884-022-04526-0 (PMC8919632; doi:10.1186/s12884-022-04526-0)
Supplement: Supplementary file 1 — Additional file 1: Supplemental Table 1. Intra-class correlation coefficients of intra- and inter-observer agreement at six ROIs. [file 12884_2022_4526_MOESM1_ESM.docx]

Supplemental Table 1. Intra-class correlation coefficients of intra- and inter-observer agreement at six ROIs.

| ROIs | Intra-observer  agreement(95%CI) | p | Inter-observer  agreement (95%CI) | p |
| --- | --- | --- | --- | --- |
| Anterior cervical lip | |  |  |  |
| Inner | 0.948（0.894-0.975） | <0.001 | 0.885(0.775-0.943) | <0.001 |
| Middle | 0.901（0.803-0.952） | <0.001 | 0.903（0.807-0.953） | <0.001 |
| Outer | 0.915（0.829-0.958） | <0.001 | 0.859（0.663-0.937） | <0.001 |
| Posterior cervical lip | |  |  |  |
| Inner | 0.933（0.864-0.967） | <0.001 | 0.868（0.742-0.935） | <0.001 |
| Middle | 0.887（0.777-0.945） | <0.001 | 0.872（0.749-0.937） | <0.001 |
| Outer | 0.924（0.847-0.963） | <0.001 | 0.909(0.819-0.956) | <0.001 |
