# Supplementary figures and images for: Clinical application of cervical shear wave elastography in predicting the risk of preterm delivery in DCDA twin pregnancy
Source: BMC Pregnancy Childbirth. 2022 Mar 14;22:202. doi: 10.1186/s12884-022-04526-0 (PMC8919632; doi:10.1186/s12884-022-04526-0)

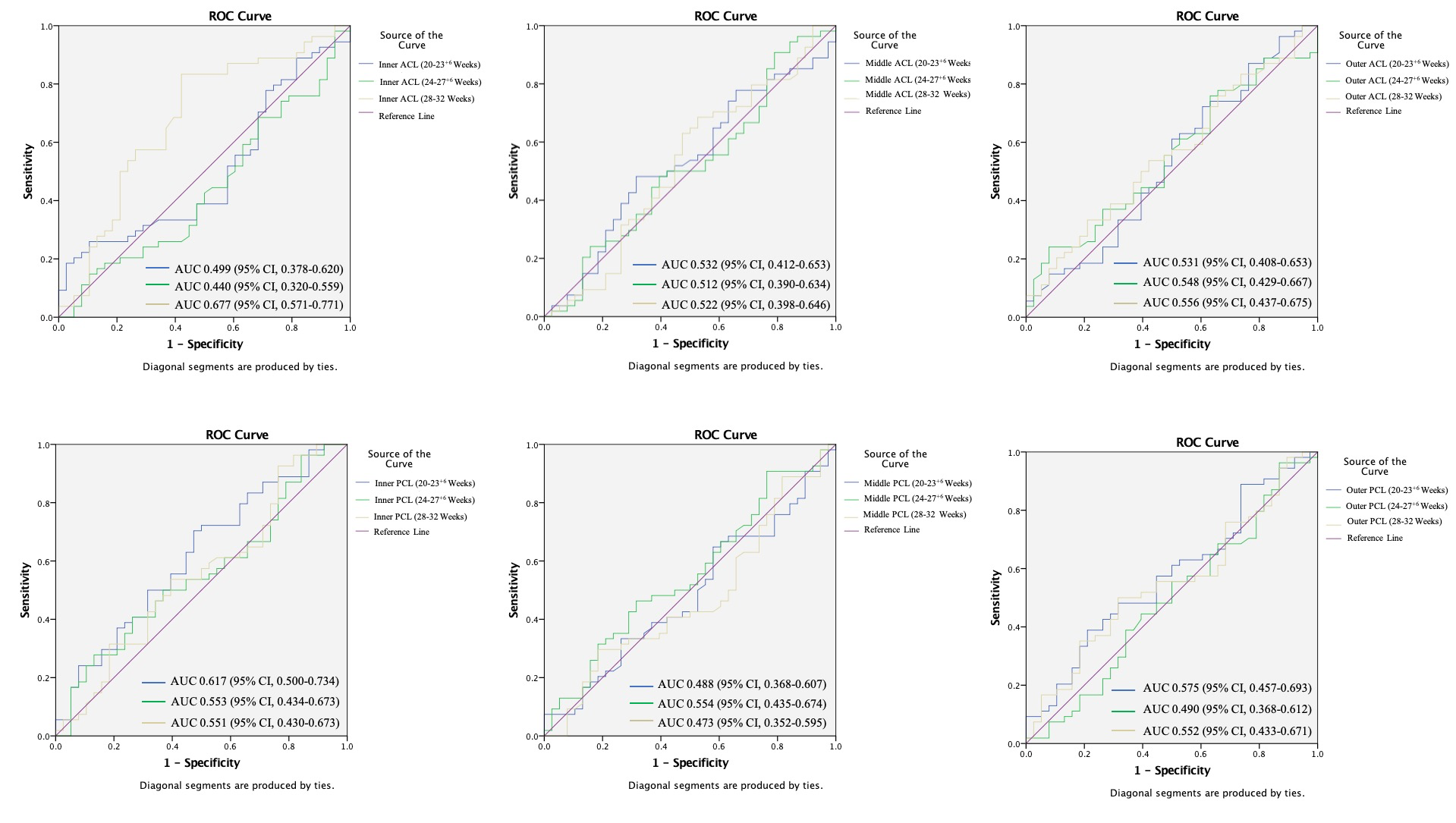

Supplement: Supplementary file 2 — Additional file 2: Supplemental Figure 1. The ROC curves of the mean SWE value of the different cervical ROIs. [file 12884_2022_4526_MOESM2_ESM.tif]

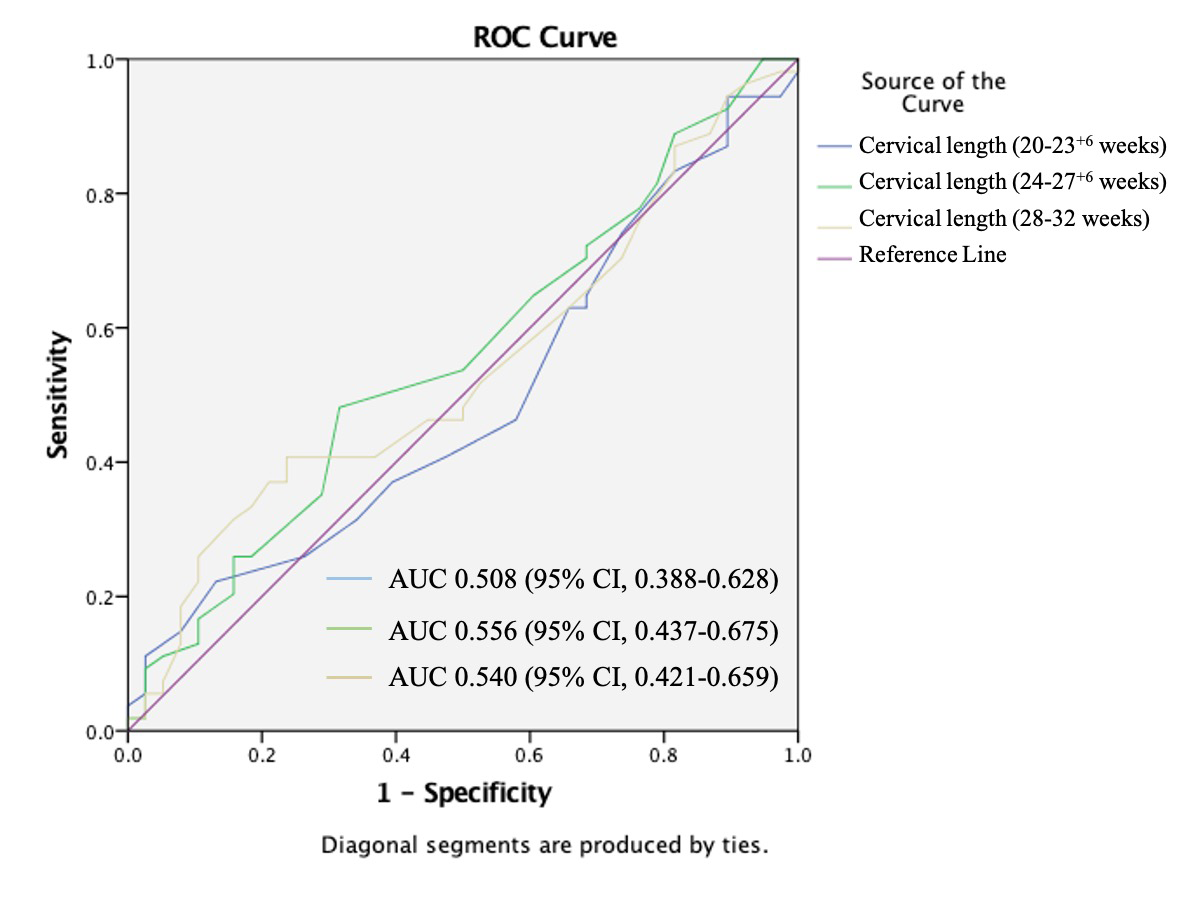

Supplement: Supplementary file 3 — Additional file 3: Supplemental Figure 2. The ROC curves of the cervical length. [file 12884_2022_4526_MOESM3_ESM.tif]
